# The control strategies for *E. coli* O157:H7 in food processing at the physical, chemical and biological levels

**DOI:** 10.3389/fmicb.2025.1598090

**Published:** 2025-06-05

**Authors:** Chenggong Xu, Zhongxiang Xin, Ruishen Yu, Jiaxin Li, Wenjia Dan, Jiangkun Dai

**Affiliations:** ^1^School of Life Science and Technology, Shandong Second Medical University, Weifang, China; ^2^Department of Orthopedics, Affiliated Hospital of Shandong Second Medical University, Weifang, China

**Keywords:** *E. coli* O157:H7, control strategies, food processing, infectious diseases, food safety and health

## Abstract

In recent years, the infectious diseases caused by pathogenic microorganisms have become one of the most prominent public health issues, which seriously endangers people’s lives and leads to significant economic losses. Studies have shown that the Shiga toxin produced by *Escherichia coli* O157:H7 (*E. coli* O157:H7) can cause severe diseases, such as hemorrhagic colitis, diarrhea, hemolytic uremic syndrome, etc. For the purpose of improving people’s health level and quality of life, it is quite important and necessary to further deepen the research on the antibacterial methods for pathogenic bacteria. In this work, we mainly summarized the control strategies for *E. coli* O157:H7 in food processing from the physical, chemical and biological levels, and summarized their own antibacterial mechanisms as well as the advantages and weaknesses. In general, physical methods are effective in eliminating *E. coli* O157:H7, but some are costly, complex, and may compromise food quality. Chemical methods, such as acidic preservatives and chlorine-based disinfectants, can also pose health risks with long-term and excessive use. In contrast, biological methods, although somewhat expensive, tend to provide safer and more environmentally friendly approaches with effective antimicrobial effects.

## 1 Introduction

Food is of vital importance to human survival and development as it can supply energy and nutrients to maintain life activities, and it is crucial to ensure high food safety while providing adequate nutrition. By virtue of the advantages that can be consumed without additional preparation such as heating or washing, the ready-to-eat foods have seen an increasing demand in recent years (Zhang Y. et al., 2024; Naseem et al., 2025). So, it is particularly important and necessary to adopt appropriate strategies to eliminate pathogenic bacteria in food processing.

**FIGURE 1:**
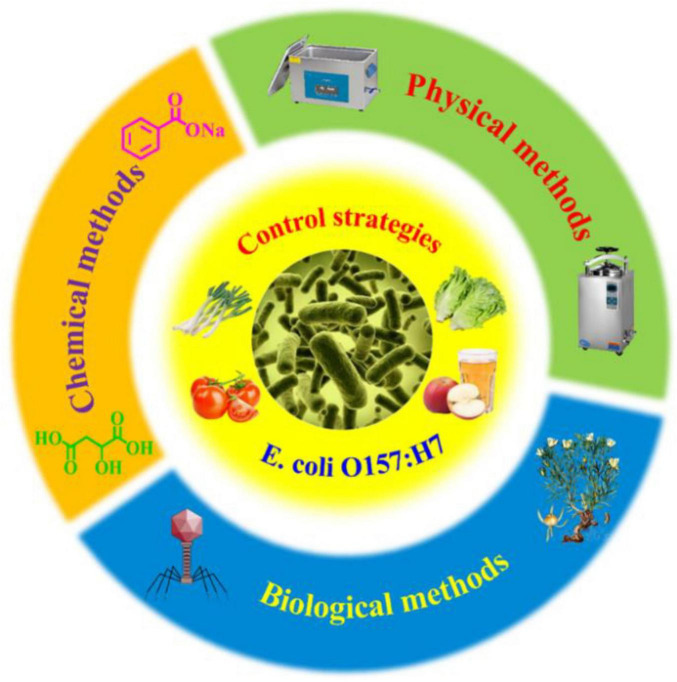
The control strategies for *E. coli* O157:H7 in food processing from the physical, chemical and biological levels.

As the most typical serotype of *Enterohemorrhagic Escherichia coli* (*EHEC*), *E. coli* O157:H7 can cause severe hemorrhagic colitis, diarrhea, and hemolytic uremic syndrome by producing Shiga toxin (Dhital et al., 2024; Jing et al., 2024; Wang et al., 2024). Statistics showed that about 2.8 million people can be infected with Shiga toxin-producing *Escherichia coli* (STEC) each year, and the resulting cases of Hemolytic Uremic Syndrome in Children (STEC-HUS) were as high as 3,890. In addition, contaminated food has also been confirmed to play an important role in infectious disease outbreaks, and there were several pandemics in 2018–2019 alone (Jones et al., 2023). Given the rapid growth, high survival rate, and strong infectivity of *E. coli* O157:H7, it is particularly necessary to adopt effective physical, chemical, and biological strategies in food processing to further improve food safety and safeguard public health (Figure 1).

## 2 Control strategies

### 2.1 Physical methods

In order to eliminate *E. coli* O157:H7 in food processing, a variety of physical control strategies have been proposed, such as new packaging and heat treatment (Oluwarinde et al., 2023). Taking the new packaging method as an example, the common vacuum packaging method is to put foods into a film with low O_2_ permeability, and then extract the air inside and place it in a vacuum sealed environment (Zhang P. et al., 2022). As for modified atmosphere packaging, the air in the package is usually replaced with a gas mixture composed of N_2_ and CO_2_ in a specific ratio to create a microenvironment with low O_2_ concentration, thereby inhibiting the biochemical reactions and microbial growth, and ultimately extending the shelf life of packaged food (Sun et al., 2022).

**FIGURE 2:**
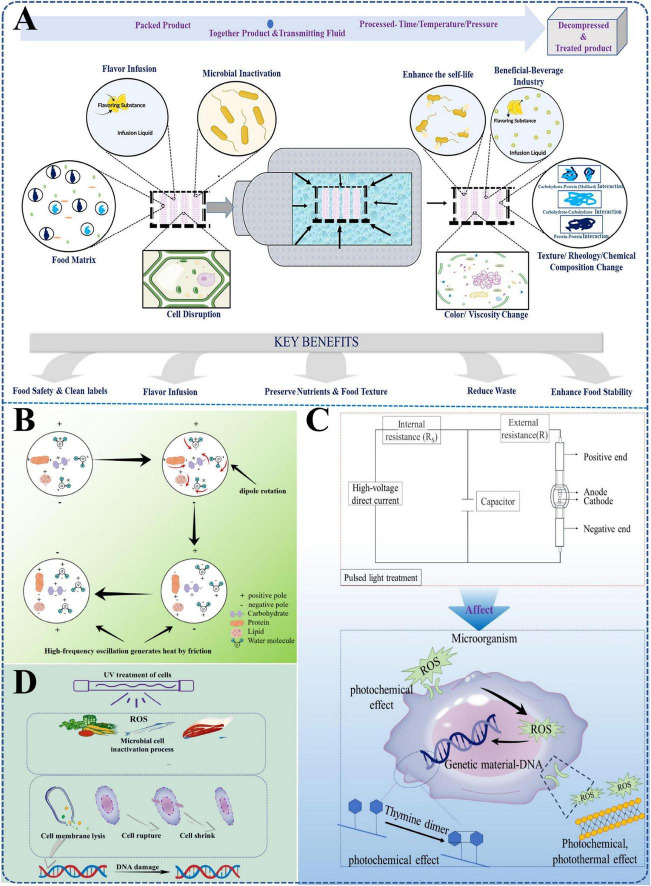
Schematic diagrams of several typical physical antibacterial strategies. **(A)** high-pressure processing. Reproduced with permission (Goraya et al., 2024). Copyright 2024, Taylor & Francis. **(B–D)** microwave heating, pulsed light and ultraviolet radiation. Reproduced with permission (Zhang Y. et al., 2024). Copyright 2024, Taylor & Francis.

As for traditional heat treatment methods, they are indeed effective in killing bacteria and other pathogens. However, during this period, high temperatures can also alter the chemical and physical properties of the food itself, and some heat-sensitive vitamins and enzymes may be destroyed, thus leading to a reduction in taste, color, aroma and nutritional value (Kim and Song, 2023). On this basis, a variety of high-performance sterilization technologies including high-pressure processing, microwave heating, pulsed light and ultraviolet radiation have been developed in recent years (Figure 2; Erdogdu et al., 2018).

#### 2.1.1 Emerging thermal technologies

Emerging thermal technologies, such as microwave heating, radio frequency, ohmic heating and superheated steam, have all shown favorable antimicrobial effects in food processing. As for microwave heating, its mechanism lies in the water can produce friction through dipole rotating under microwave, thereby producing a large amount of heat in food and achieving the sterilization effect (Dutta et al., 2024; Kim et al., 2024). Studies have shown that the water activity in food has a significant impact on the microwave heating effect, and foods with high water content tend to exhibit better antibacterial effects (Song and Kang, 2016). Siguemoto et al. (2018) used microwaves to treat apple juice inoculated with *E. coli* O157:H7 and found that the content of pathogenic bacteria could be reduced to the standard recommended by the US Food and Drug Administration (FDA). Compared with traditional heat treatment methods, although microwave heating showed high sterilization efficiency and little impact on the quality of the food itself, it may also lead to uneven sterilization results due to the inhomogeneity of microwave radiation and the difference in water content. So, there are still some challenges to completely eliminate pathogenic bacteria from different kinds of food (Chang et al., 2022).

Similar to microwave heating, radio frequency is also a physical technique that inactivates bacteria using heating effect of medium, and its mechanism involves thermal shock (Tong et al., 2022). Specifically, when food is exposed to an alternating electric field, dipole molecules such as water will generate intermolecular friction through self-calibration, and release a large amount of heat to exert sterilization effects. It is noteworthy that unlike other emerging thermal technologies, radio frequency can effectively eradicate bacteria in foods with both high and low water content (Jiao et al., 2014). Moreover, low-moisture foods are susceptible to contamination with *E. coli* O157:H7, which makes this technology a significant advantage in food quality control.

Ohmic heating is a technique that utilizes the electrical resistance of food itself to generate heat and raise the temperature (Hradecky et al., 2017). Specifically, when an electric current flows through food, electrons will collide with neutrons, atoms, and other electrons, thereby releasing heat and killing bacteria. Studies have shown that ohmic heating is generally not limited by the size and shape of food itself, which gives it the obvious advantage of fast and uniform heating in food sterilization (Çokgezme et al., 2021). However, some potential threats still require attention. Tian et al. (2021) utilized the high-throughput Illumina HiSeq 2000 mRNA sequencing platform to analysis the genetic changes of *E. coli* O157:H7 after ohmic heating. The results showed that the pathogenic bacteria can obtain up to 516 gene expression changes after ohmic heating, and the expression of most virulence genes was significantly upregulated, which may significantly improve the pathogenicity of the surviving *E. coli* O157:H7 (Tian et al., 2021).

Superheated steam refers to the steam with higher temperature and enthalpy than conventional or saturated steam. When the superheated steam condenses on the surface of food, a large amount of heat can be transferred, thereby causing the temperature of the food to rise and killing bacteria efficiently (Rana et al., 2022). Studies have shown that the use of superheated steam in food processing can save time and energy, while eliminating the need for chemical preservatives, which may provide an environmentally friendly antibacterial solution (Zhang H. et al., 2024). Ban and Kang (2018) treated cherry tomatoes and oranges inoculated with *E. coli* O157:H7, *Salmonella typhimurium*, and *Listeria monocytogenes*, and found that the superheated steam can be more effective in inactivating pathogenic bacteria than saturated steam, and no significant decrease in food appearance and nutrition was observed (Ban and Kang, 2018).

#### 2.1.2 Ultrasonic cleaning

Cleaning is an indispensable part in food processing, especially in the processing of vegetables and fruits, as it can effectively remove residual contaminants such as soil, insects, pesticides, and microorganisms. At the same time, ultrasonic cleaning has also received widespread attention due to its high environmental friendliness and safety. Studies have demonstrated that ultrasonic can enhance cleaning efficacy and notably increase the permeability of fruit and vegetable cells, which leads to enhanced drying and dehydration outcomes, and ultimately extending the shelf life of the produce (Yu et al., 2024). Besides, studies have also confirmed that ultrasonic cleaning can promote the accumulation of phenols, carotene and ascorbic acid in plant tissues, and improve their nutritional value effectively (Zhou et al., 2022).

Although ultrasonic cleaning performs well in food processing, its sterilization efficiency is usually not high enough when used alone. Iorio et al. (2019) found that the *E. coli* O157:H7 in almond milk was reduced by 1.31 log CFU/mL after treating with 20 kHz, 130 W ultrasound for 6 min. Bonah et al. (2021) used 20 kHz, 300 W ultrasound to treat pork samples for 30 min and found that the *E. coli* O157:H7 was reduced by only 3.8 log CFU/g. So, there have been some reports of combining ultrasonic cleaning with other technologies to further improve its sterilization effect. Li et al. (2024) found that the combined treatment of *E. coli* O157:H7 with high-frequency ultrasound and sodium hypochlorite can obtain a significantly enhanced antibacterial effect, and the bacterial reduction can be as high as 6 log CFU/mL. As for the mechanism, it mainly involves that reactive oxygen species (ROS) can easily pass through the cell membrane barrier or diffuse through pores under the acoustic pore effect of high-frequency ultrasonic, thereby causing severe bacterial oxidative damage (Li et al., 2024).

#### 2.1.3 Ionizing radiation

Ionizing radiation, including X-rays, gamma rays, and electron beams, has also been used in food processing, and its mechanism involves destroying the protein and DNA structures of microorganisms, thereby killing bacteria, parasites and fungi effectively (Fernandes and Prakash, 2020). However, there is currently some concern among consumers about the potential risks of ionizing radiation, which may be attributed to the unconscious association of its name and nuclear radiation.

As a special type of ionizing radiation, nuclear radiation is widely used in industry, medicine and scientific fields, and they do pose some potential health risks. However, in the food industry, the Joint FAO/IAEA/WHO Expert Committee has stated that the treatment of food with a dose of 10 kGy does not generally have adverse effects on its safety and nutritional composition (Fatemi and Frank, 1999). Therefore, ionizing radiation possesses high application potential in food sterilization. Osaili et al. (2018) treated tahini halwa containing *E. coli* O157:H7 with gamma rays and found that the D_10_-value was as low as 1.83 KGy, which also confirmed this physical technology can be successfully used for food disinfection.

#### 2.1.4 High-pressure processing

As a non-thermal food processing technology, high-pressure processing generally uses water or oil as the pressure transfer medium to treat food sealed in a container with high pressure (Jeon et al., 2024). Studies have shown that high-pressure processing can effectively eliminate most microorganisms, including bacteria, mold and yeast, and has little effect on the color, aroma, taste and nutritional of the food itself (Khan et al., 2017). Zhou et al. (2016) treated ground beef containing *E. coli* O157:H7 under 400 MPa for 5 cycles of 3 min at 25°C, and found that the reduction of pathogenic bacteria was as high as 5-log. As for its mechanism, some studies have confirmed that the inactivation of microorganisms usually involves the changes in bacterial genes, biochemical reactions, cell membranes and cell morphology under high pressure (Goraya et al., 2024). Therefore, with the combined effect of these factors, high-pressure processing can obtain a satisfactory sterilization effect and ensure high safety in food processing.

#### 2.1.5 Ultraviolet radiation

Ultraviolet radiation has been widely used in bacteria elimination due to its high cost performance, easy operation, no irritating by-products generation and wide disinfection range. Its mechanism involves DNA damage, that is, ultraviolet light can directly induce the formation of dimerization of pyrimidine bases in bacterial nucleic acid, thus inhibiting the DNA replication process and leading to bacteria death (Kim and Kang, 2020). Besides, some studies also confirmed that ultraviolet radiation can effectively reduce pathogenic bacteria contamination, while has negligible impact on the quality of food (Usaga and Worobo, 2018). Donsingha and Assatarakul (2018) also found that *E. coli* O157:H7 is highly sensitive to ultraviolet light, and its population can decrease the most in coconut juice after irradiation treatment. Overall, with the rapid development of emerging high-efficiency ultraviolet disinfection technology, ultraviolet radiation possessing high radiation stability and longevity is expected to provide more reliable and durable disinfection in food processing.

#### 2.1.6 Pulsed light

As a physical sterilization technology, pulsed light generally uses inert gasses such as xenon to release pulsed light with a wide spectrum and high energy in a time as short as tens to hundreds of microseconds (John and Ramaswamy, 2018). With this technology, the light energy equivalent to hundreds of thousands of times the intensity of sunlight reaching the ground can be released within a quite short period, and realize the elimination of harmful microorganisms including viruses, fungi and bacteria. Its mechanism involves the changes of DNA structure under pulsed light, accompanied by the destruction of cell membranes and proteins, thus leading to cell death and achieving a highly efficient bactericidal effect (Nicorescu et al., 2013). Xu et al. (2013) used pulsed light to treat green onions containing pathogenic bacteria and found that the inactivation effect of more than 4 log CFU/g can be obtained after irradiation of dry and water-soaked samples for 5 and 60 s, respectively. However, it should also be noted that although pulsed light can exhibit good sterilization performance, its equipment is generally expensive and bulky, which may limit its wider application in the food industry.

#### 2.1.7 Cold plasma

Cold plasma, as one kind of non-thermal antimicrobial method, has received great attention by virtue of its high safety, low cost, and the ability to be used for the treatment of in-package food. Studies have shown that cold plasma generally contains a large amount of charged particles, such as OH^–^, H_3_O^+^, ROS, RNS, excited O_2_, and N_2_, as well as ultraviolet rays, which makes it effective in killing both bacteria floating in the air and cling to the surface of food (Alaguthevar et al., 2024). Niemira (2012) treated almonds containing *Salmonella* and *E. coli* O157:H7 at 6 cm spacing for 20 s with cold plasma and found that the amount of both pathogens was significantly reduced, and the reduction in *E. coli* O157:H7 was up to 1.34 log CFU/mL. Min et al. (2017) used cold plasma to treat bulk Romaine lettuce in containers and found that the amount of *E. coli* O157:H7 on leaf samples can be reduced by 0.4–0.8 log CFU/g, while no significant impact on its physical and biological properties, which makes it a promising post-packaging sterilization technology in food processing. Although cold plasma sterilization has shown good performance in the field of food processing, its cost and requirements for operation technique are relatively high. Therefore, it is necessary to comprehensively consider its advantages and limitations in practical application, and determine whether to use this technology according to the specific processing purpose.

### 2.2 Chemical methods

As a chemical additive, preservatives can greatly inhibit the life activities of microorganisms such as bacteria, molds and yeasts, thereby keeping food fresh and extending its shelf life. At present, a variety of acidic preservatives and chlorine-containing disinfectants have been proposed, such as peracetic acid, organic acid, sodium benzoate and sodium hypochlorite, and most of them have exhibited good performance in eliminating foodborne pathogens (Yoder et al., 2012).

#### 2.2.1 Acid preservatives

In 1902, Freer and Novy firstly demonstrated that peracetic acid featured with excellent bactericidal properties. The subsequent studies showed that peracetic acid can keep stable in the presence of organic compounds, and was also strongly bactericidal when used at low concentrations (Gurtler et al., 2022). As for the antibacterial mechanism, it involves that peracetic acid, with strong oxidizing property, can cause severe damage to cell walls and membranes, thereby leading to bacteria death. Hilgren and Salverda (2000) used peracetic acid to treat fresh-cut-vegetable process water and found that the acid preservative at concentrations as low as 0.05–0.50% can exhibit strong eliminating properties on yeast and mold. Kalchayanand et al. (2024) also found that after treating beef contaminated with *E. coli* O157:H7 and *Salmonella* with peracetic acid, the amount of both pathogens was significantly reduced. However, it should also be noted that although peracetic acid has a good sterilization effect, it tends to release pungent odor during use. Therefore, more attention should be paid to its potential impact on the human body and the environment in food processing.

Organic acids, including acetic acid, lactic acid, butyric acid, citric acid, and malic acid, can also be used as bactericides in food processing due to their good antimicrobial properties and high food safety (Sharma and Lee, 2025). Some studies have confirmed that the antibacterial mechanism of organic acids involves energy competition, the changes in bacterial membrane permeability and intracellular osmotic pressure, as well as inhibition of biomolecular synthesis (Bai et al., 2022). In the presence of organic acids, the acidic molecules can penetrate through bacterial wall and accumulate inside, resulting in a significant increase in intracellular osmotic pressure. Under this condition, some precursor compounds and cofactors essential for bacterial growth will be released into the external environment to maintain the balance of osmotic pressure inside and outside cells, which greatly inhibits their growth and reproduction (Yoon et al., 2024). In addition, the high concentrations of organic acids in cells can also interfere with or even prevent the DNA synthesis process, which affects their subsequent life activities and ultimately leads to bacteria death (Rasch, 2002; Pieterse et al., 2005). Wang et al. (2019) treated lettuce containing *E. coli* O157:H7 and *Listeria monocytogenes* with acetic and lactic acid, and found that the mixture of the two organic acids can exhibit the most significant antibacterial effect, and no additional loss of polyphenol content was observed at acetic acid concentrations below 0.8%.

As the sodium salt of benzoic acid, sodium benzoate is also a commonly used food preservative additive. By virtue of its high food safety, it has been listed as a Generally Recognized as Safe (GRAS) additive by the FDA (Thomazini et al., 2023). Studies have shown that the antibacterial effect of this preservative can be greatly affected by environmental pH (López-Malo et al., 2005). Specifically, in the pH ranging from 2.5 to 4.0, it often exhibits good antibacterial effects, and the bactericidal effect can be significantly enhanced as the acidity increases, while tends to lose antibacterial properties under alkaline conditions (Chen and Zhong, 2018). Chen and Zhong (2018) used sodium benzoate to treat cherry tomatoes infected with *E. coli* O157:H7 under different pH conditions, and found that this chemical preservative can exhibit better antibacterial effect as the pH decreased. Sodium benzoate generally possesses good antibacterial performance and high biosafety, but it should also be noted that the long-term or excessive intake may cause severe damage to the liver and kidneys (Piper and Piper, 2017). Therefore, it is crucial to ensure that the concentration and dosage of sodium benzoate added are within safe limits in food processing.

#### 2.2.2 Chlorine-containing disinfectants

**FIGURE 3:**
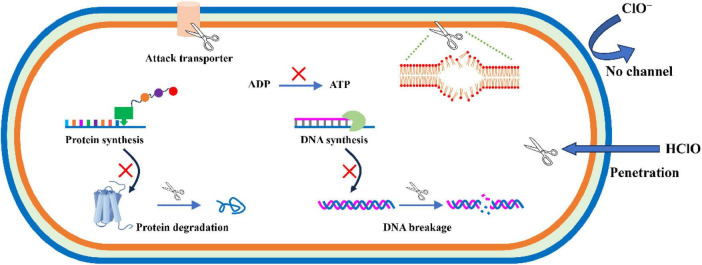
Schematic diagram of the effect of HClO on *E. coli* O157:H7.

Chlorine-containing disinfectants refer to antiseptics that can produce hypochlorous acid with antibacterial activity after dissolving in water (Yi et al., 2024). Among them, sodium hypochlorite has attracted widespread attention by virtue of its excellent bactericidal properties (Figure 3). After sodium hypochlorite was ionized in water and converted into hypochlorous acid, it can cause severe damage to bacterial cell membranes (Fukuzaki, 2006). Besides, as a kind of strong oxidant, hypochlorous acid can also react irreversibly with enzymes and structural proteins containing sulfur and heme, thereby causing damage to the respiratory chain (Schaffer et al., 2009). In addition, some studies have confirmed that hypochlorous acid can also cause metabolic dysfunction and ultimately lead to bacteria death (King et al., 2004).

Chlorine dioxide, as a gaseous oxidant, is also highly effective in inactivating foodborne pathogens on the surface of the food. Visvalingam and Holley (2018) exposed beef containing *E. coli* O157:H7 to 200 ppm chlorine dioxide for 4 days and found that the reduction of pathogenic bacteria was up to 2.8 log CFU/g, which was even better than the effects after treatment with sodium hypochlorite and peracetic acid under the same conditions. Overall, chlorine-containing disinfectants have shown good application potential in food processing due to their excellent antibacterial properties. However, it should also be noted that this antibacterial agent is often highly irritating and lack sufficient chemical stability, and some chlorides such as chloramines and chlorobenzene can be produced after disinfection, which may pose a risk of cancer and distortion (Ferraris et al., 2005). Therefore, when using chlorine-containing disinfectants for food sterilization, the standard operating procedures should be strictly followed to ensure that the disinfection effect can be obtained while minimizing its adverse effects on the human body and the environment.

### 2.3 Biological methods

With the development of society, people have put forward new and higher requirements for food safety. Biological control strategies, as a kind of efficient and safe antibacterial technologies, have been increasingly favored in food industry. Therefore, a variety of biological antibacterial agents such as bioprotective microorganisms, plant-derived natural compounds and bacteriocins were proposed to combat *E. coli* O157:H7 in food processing, and most of them exhibited good antimicrobial effects while negligible impact on the nutrition of the food itself, which makes them a good choice in food processing (Puligundla and Lim, 2022).

#### 2.3.1 Microbial control

**FIGURE 4:**
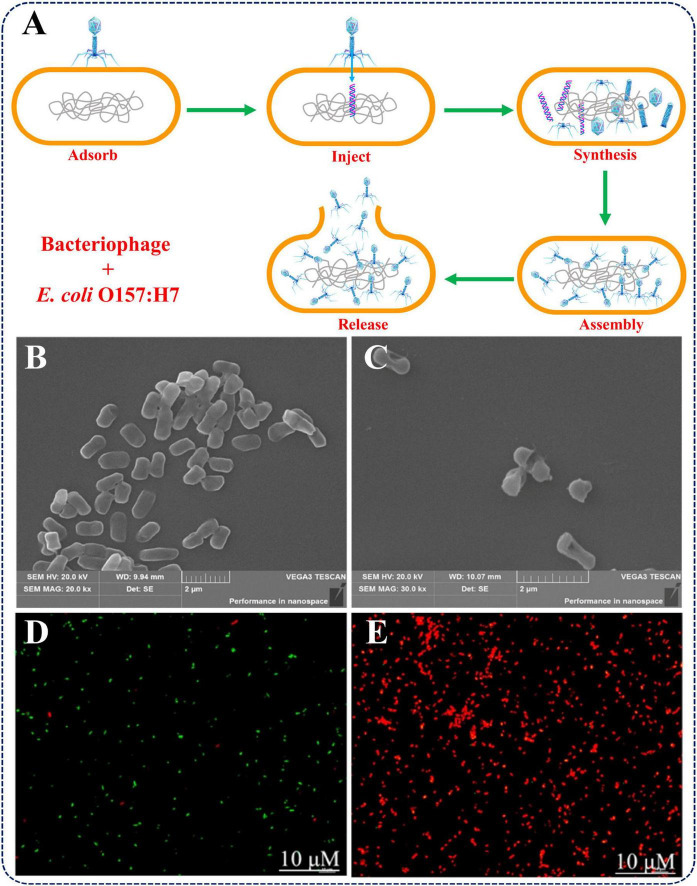
**(A)** The antibacterial mechanism of bacteriophage. **(B,C)** SEM images of *E. coli* O157:H7 before and after treatment with zp37, and **(D,E)** fluorescence changes with SYTO9/PI incubation. Reproduced with permission (Yi et al., 2021). Copyright 2021, Elsevier.

Microbial control refers to the strategy that using protective microorganisms, such as bacteriophages, probiotics and antagonistic bacteria with antagonistic activity to inhibit or even inactivate pathogenic bacteria (Lin et al., 2023). Bacteriophages, as one type of virus that parasitizes bacteria, can take advantage of the host’s DNA replication and protein synthesis systems to reproduce themselves, and lyse the bacteria ultimately (Figure 4A). Given their negligible risk to humans, bacteriophages can also be used for the elimination of specific bacteria in food processing (Abedon, 2009). Coffey et al. (2011) used a specific bacteriophage to treat broth containing *E. coli* O157:H7 and found that the number of pathogens can be significantly reduced under a variety of environmental conditions. Gencay et al. (2016) used bacteriophage M8AEC16 to treat Turkish raw meatballs inoculated with *E. coli* O157:H7 and found that the reduction of live pathogens can reach up to 1.85 log CFU/g.

Probiotics are microorganisms that can grow and reproduce in the human intestines, and plays important roles in maintaining the balance of intestinal flora and promoting the health of digestive system (Lau and Quek, 2024). Currently, probiotics represented by *Lactobacillus acidophilus* have also been used as food additives and showed good antibacterial effects. Wang et al. (2023) found that the *Lactiplantibacillus plantarum* Lac16 can significantly restrict the growth of *E. coli* O157:H7, and its mechanism involves inhibiting the formation of bacteria biofilm and the expression of mRNA related to virulence traits. Xing et al. (2023) found that *Lactobacillus acidophilus* AD125 also possessed strong antibacterial activity and satisfactory gastrointestinal tolerance, which makes AD125 a good candidate in preventing intestinal diseases caused by relevant pathogenic bacteria.

Antagonistic bacteria also play a significant role in food processing, where they can exert their antimicrobial effects by producing antimicrobial metabolites or competing with pathogenic bacteria for nutrients and space (Haidar et al., 2016). Olanya et al. (2020) found that the *Bdellovibrio bacteriophage* 109, a predator of Gram-negative bacteria, can effectively alleviate the contamination of lettuce and carrots by *E. coli* O157:H7. Koo et al. (2015) also found that *Leuconostoc* tend to exert its antibacterial effect by producing active antibacterial substances such as organic acids, so the growth of *E. coli* O157:H7 can be significantly inhibited by adding cell-free supernatant (Koo et al., 2015). More importantly, as a biological control agent, antagonistic bacteria usually possess high environmental friendliness and antimicrobial specificity, thus significantly reducing environmental pollution and damage to ecosystems.

#### 2.3.2 Natural compounds

Natural plant products such as flavonoids, alkaloids, phenols, tannins, and saponins are a class of compounds with antibacterial activity, and they all play important roles in food sterilization (Kanmani Bharathi and Prakash, 2025). Sewlikar et al. found that the *Quillaja saponaria* aqueous bark extracts containing bioactive polyphenols, tannins, and tri-terpenoid saponins can bring damage to the cell membrane of *E. coli* O157:H7 and reduce its colony number significantly after 1 h of treatment (Sewlikar and D’Souza, 2017). Issazadeh et al. (2021) treated *E. coli* O157:H7 with an alcohol extract of *Spinacia oleracea* leaves rich in phenolic compounds and alkaloids, and also found that it could cause serious damage to the cell walls of pathogenic bacteria and effectively inhibit their growth and reproduction.

Trans-cinnamaldehyde, as a natural antibacterial compound, can also cause bacteria death. Baskaran et al. (2010) treated *E. coli* O157:H7 inoculated apple juice and cider with trans-cinnamaldehyde and found that concentrations as low as 0.025% of the compound were sufficient to achieve satisfactory bactericidal effects. Olszewska et al. (2020) found that trans-cinnamaldehyde can effectively disrupt cell morphology and reduce bacterial metabolic activity by inhibiting cell membrane formation.

Gallic acid and thymol also showed strong antimicrobial effects. Zhang C. et al. (2024) treated *E. coli* O157:H7 with gallic acid screened from plant-derived small molecules, and found that this natural compound can inhibit the formation of biofilm effectively, and then exert a good antibacterial effect. In addition, as another natural compound, thymol has also been confirmed by Guarda et al. (2011) and Zhang et al. (2022) to have satisfactory antibacterial effect against *E. coli* O157:H7, and the combination with gallic acid can produce more efficient synergistic antibacterial effect.

Flavonoids and alkaloids, as organic compounds widely existing in plants, have also been proven to have good antibacterial properties. Vikram et al. (2010) found that flavonoids such as naringenin, quercetin, sandalin and apigenin, all possessed obvious inhibitory effects on the formation of *E. coli* O157:H7 biofilm as well as the communication among bacteria. Besides, the isopentadienated flavanones isolated from *Macaranga tanarius* have also been proven to have antibacterial activity due to the presence of flavonoid skeleton and ester groups. What’s more, some alkaloids also exhibited strong antibacterial properties. Lee et al. (2017) found that both harmaline and norharmane extracted from the seed of *Peganum harmala L* can restrict the formation of *E. coli* O157: H7 biofilms. At the same time, the virulence of the pathogen was reduced in animal experiments, and the lifespan of infected nematode model can be extended.

In general, natural compounds represented by flavonoids, alkaloids, phenols, tannins, and saponins have exhibited satisfactory antimicrobial properties and biosafety. However, most of them are generally expensive, poorly water-soluble, and even have the potential to react with food ingredients, which makes their wider application in food processing still challenging.

#### 2.3.3 Bacteriocin

Bacteriocins are proteins or peptides produced by a specific type of bacteria in a multi-microbial environment, and can inhibit or even kill the competing bacteria by interfering with their life activities (Simons et al., 2020). For instance, the bacteriocins can disrupt and make pores in bacterial cell membranes, causing the cellular proton gradient to be out of balance, which hinders normal cell metabolism and leads to bacteria death (Devlieghere et al., 2004). Studies have shown that bacteriocins, as a natural product, are generally harmless to the human body and have negligible effect on the physical and chemical properties of the food itself (Yang et al., 2024). In addition, this compound also has excellent biodegradability and can be effectively eliminated by intestinal proteases, thus being assessed as GRAS (Javed et al., 2011).

By virtue of the good antibacterial effect and biosafety, the bacteriocins can also be used for eliminating *E. coli* O157:H7 in food processing. Yi et al. (2021) treated bean sprouts containing *E. coli* O157:H7 with antimicrobial peptide zp37 for 7 days and found that the activity of this pathogen was reduced by up to 94.7%. As for its antimicrobial mechanism, subsequent studies have shown that zp37 can effectively induce bacterial membrane depolarization, resulting in DNA aggregation and loss of function after zp37 enters the cytoplasm and binds to bacterial DNA, and ultimately leads to bacteria death (Figures 4B–E). Öncül and Yildirim (2019) also demonstrated that enterococcin KP and lactococcin BZ, whether used alone or in combination, can exhibit strong bactericidal effects against *E. coli* O157:H7 in milk, and are expected to be applied in pathogen control in the dairy industry.

Although bacteriocins exhibit good bacteriostatic effects in the laboratory, their widespread application in food industry is still subject to some limitations. Specifically, the diffusion of bacteriocins in solid foods is often restricted, making it difficult to thoroughly cover the food. Besides, some bacteriocins can only inhibit specific microorganisms and their sterilization effects are greatly affected by the external environment. What’s more, after prolonged exposure, the bacteriocins may promote resistance to some bacteria, and weaken or even completely eliminate their bacteriostatic effects (Bastos Mdo et al., 2015).

## 3 Conclusion

In summary, most physical, chemical and biological strategies all play an indispensable role in the elimination of *E. coli* O157:H7 in food processing and jointly promote food safety, but still encounter some challenges. Some physical methods general costly and complex to operate, and have the risk of affecting food quality. As for chemical methods, the widely used acidic preservatives and chlorine-containing disinfectants may also cause harm to our health after long-term and excessive use. In contrast, biological methods seem to be healthier and more environmentally friendly while delivering good antimicrobial results. Moreover, it can be estimated that with the continuous development of the national economy and people’s attention to food safety, the role of biological methods in the food industry will gradually increase, regardless of its higher price.
